# Paradoxical and Unmasking HIV Immune Reconstitution Inflammation Syndrome in Antiretroviral-Naïve Pregnant Women: A Prospective Cohort Study

**DOI:** 10.7759/cureus.52989

**Published:** 2024-01-26

**Authors:** John K Muthuka, Everlyn M Nyamai, Kelly Oluoch, Charles Maibvise, Rosemary Nabaweesi

**Affiliations:** 1 Epidemiology, Public Health, and Biostatistics, Jomo Kenyatta University of Agriculture & Technology, Nairobi, KEN; 2 Public Health Sciences, Kenya Medical Training College, Nairobi, KEN; 3 Nursing, Kenya Medical Training College, Nairobi, KEN; 4 Pharmacy, Kenya Medical Training College, Nairobi, KEN; 5 Nursing, University of Eswatini, Mbabane, SWZ; 6 Health Policy, Meharry Medical College, Nashville, USA

**Keywords:** pregnancy, antiretroviral therapy, hiv aids, clinical parameters, immune reconstitution syndrome

## Abstract

Background and aims: Following antiretroviral therapy (ART) initiation, HIV-associated immune reconstitution inflammatory syndrome (IRIS), indicated by an array of opportunistic infections, may occur, presenting as either paradoxical, a worsening of a previously treated infection, or unmasking, a flare-up of an underlying, previously undiagnosed infection. The impact of ART as the backbone of HIV treatment and prevention has prolonged the survival of people living with HIV. In pregnancy, benefits have been shown by slowing HIV progression and preventing perinatal transmission; however, there have been risks of adverse reactions with ART, including immune responses to both the fetus and mother. This study sought to estimate the incidence of HIV-IRIS cumulatively and by type either paradoxical or unmasking IRIS, determine the baseline maternal and HIV clinical markers as predictors of, and analyze the log-rank test for survival time to IRIS outcome assessed by relying on an increase in CD4 count and/or a rapid decrease in viral load.

Methods: An active records study was conducted between June 2019 and March 2020 among ART-naïve pregnant women attending the antenatal care units (ANCu) at the Kenyatta National and Mbagathi Hospitals, Nairobi, Kenya. Participants were aged between 20 and 49 years and had a confirmed HIV-positive test. To ascertain a true case of IRIS, the diagnosis was adjudicated for accuracy and consistency by an independent review committee. Plasma HIV viral load, CD4 counts, and routine laboratory evaluations (hemoglobin, white blood cell count (WBC)) were performed by each hospital's clinical laboratory. The IRIS incidence was assessed using the International Network for Studies Against HIV-Associated IRIS (INSHI) during the first three months after ART initiation. Multivariate Cox regression with IRIS as the outcome, using the SPSSSurvival package, examined the relationship between baseline maternal characteristics and HIV clinical markers before ART initiation and IRIS, and finally, decision-tree analysis for predicting IRIS was performed.

Results: From a pool of 532 ART-naïve pregnant women, 133 (25%) developed IRIS, and 97 (72.9%) were in the unmasking category. The accumulated risk of experiencing IRIS symptoms increased from week two (hazard ratio (HR)=0.0287) to week 12 (HR=3.6158). Participants with a maternal BMI (MBMI) of 25-29.9 kg/m^2^ at baseline were at a higher risk of unmasking IRIS (HR=2.478; P=0.010). The WHO-HIV clinical stages 1 and 2 skewed towards paradoxical IRIS (regression coefficients =-0.111 and -0.276 (P<0.05)), while stage 4 skewed towards unmasking IRIS (regression coefficient=0.047, HR=1.048, P=0.941). A CD4 count > 500 cells/mm^3 skewed towards unmasking IRIS (regression coefficient=0.192, HR=1.211, P=0.416), while RNA-HIV viral loads >50 copies/ml towards paradoxical IRIS (regression coefficient=-0.199, HR=0.820, P=0.360. On decision tree analysis, 85% (P=0.001) of ART-naïve pregnant women with a baseline CD4 count below 500 copies/mm^3 presented with unmasking IRIS.

Conclusion: For ART-naïve pregnant women, unmasking IRIS is the most common type, and an MBMI of 25-29.9 kg/m^2^, advanced HIV infection, a CD4 count <500 cells/mm^3, and a higher parity at baseline may be clinically useful predictors. The higher proportion of ART-naïve pregnant women experiencing unmasking as compared to paradoxical IRIS supports the need for earlier assessment based on potential predictors.

## Introduction

Immune reconstitution inflammatory syndrome (IRIS) is a clinical complication in some patients infected with HIV after starting antiretroviral therapy (ART). Immune reconstitution inflammatory syndrome is characterized by the release of proinflammatory cytokines and tissue inflammation, and it is related to a well-identified coinfection [1].

All pregnant women with HIV should initiate ART as early in pregnancy as possible, regardless of their RNA-HIV level or CD4 T lymphocyte count, to maximize their health and prevent perinatal HIV transmission and secondary sexual transmission [2]. One of the goals of ART during pregnancy is to achieve and maintain HIV viral (RNA-HIV) suppression to undetectable levels of less than 200 copies of HIV per milliliter of blood [3].

Despite the known incidence of IRIS in general after ART commencement, its estimation by IRIS type, either unmasking or paradoxical, is not well documented in the context of ART-naïve pregnant women, with research primarily focusing on the IRIS defining opportunistic infections in HIV infection. It is during pregnancy when the maternal immune system is characterized by a reinforced network of recognition, communication, trafficking, and repair; it can raise the alarm, if necessary, to maintain the well-being of the mother and the fetus. This physiologic state of pregnancy occurs regardless of HIV status [4].

Research has demonstrated a higher risk of IRIS incidence among persons living with HIV with a low CD4+ T cell count below 500 cells per cubic millimeter at baseline before ART initiation [5]. Although several aspects of IRIS pathogenesis, which include an imbalance between anti-inflammatory cytokines and pro-inflammatory cytokines, remain unclear, two clinical parameters have been related to IRIS development: (1) severe CD4+ lymphopenia at the initial presentation and (2) opportunistic diseases that lead to a dysregulated immune response (usually characterized by persistent T cell activation, which favors T cell exhaustion) [6]. Immune reconstitution inflammatory syndrome is diagnosed using the criteria from the International Network for the Study of HIV-Associated IRIS (INSHI) [7].

This study was conducted to estimate the incidence of HIV-IRIS cumulatively and by type (paradoxical or unmasking IRIS). Further, it determines baseline maternal and HIV clinical markers as predictors of and analyzes how long it took for a specific type of IRIS to occur by such predictors among ART-naïve pregnant women visiting a maternal and child healthcare clinic (MCH) for the first time and put on ART in the first trimester before assessing the specific type of IRIS outcome. To examine the complex interactions among risk factors for either a paradoxical or unmasking IRIS diagnosis, a decision tree analysis was conducted.

This article was previously posted to the medRxiv preprint server on August 22, 2023.

## Materials and methods

Study overview

The current hospital-based, active records study was conducted among ART-naïve, HIV-positive pregnant women who visited MCHs for the first time during the first trimester, prior to antenatal care services, and were diagnosed with IRIS (either unmasking or paradoxical) at the Kenyatta National and Mbagathi hospitals in Nairobi, Kenya, from June 1, 2019, to March 30, 2020. The rationale for selecting Kenyatta National Hospital was because it is the main national referral facility in Kenya, where the best specialized medical personnel would be available to offer a timely opinion on an IRIS diagnosis. On the other hand, Mbagathi Hospital was selected for its HIV comprehensive care clinic targeting pregnant women in the region, which would provide timely and reliable data for the study. The two facilities are in close proximity, and they co-share medical specialists, a reason that was crucial in ensuring knowledge was shared in validating the information for IRIS diagnosis. The study aimed at estimating the cumulative incidence of IRIS, the incidence of paradoxical and unmasking IRIS, and identifying their clinical predictors. Further, it aimed at establishing the rate at which a specific IRIS type occurred and, finally, through decision tree analysis, examining the complex interactions of risk factors for either a paradoxical or unmasking IRIS diagnosis.

Inclusion and exclusion criteria

Pregnant women who were ART-naïve and HIV-positive, aged between 20 and 49 years, diagnosed with IRIS (unmasking or paradoxical), during the first trimester, having become pregnant not later than one month at the first visit (the gestational age based on the date of the last menstrual period (LMP) concept), who were willing to participate in the study and gave consent for the utilization of their active records were included. Pregnant women who were ART-naïve and HIV-positive, aged below 20 years or above 49 years, with an unclear IRIS diagnosis, pregnant for more than one month at the first visit, and with known critical and any severe threatening conditions, including bleeding, hyperemesis gravidarum, which is excessive vomiting, spontaneous abortions or miscarriages, ectopic, and molar pregnancies, were excluded. Participants unwilling to participate in the study at the enrollment stage and declining to give consent for the utilization of their active records were also excluded.

Sample size calculation

The sample size was determined based on the following formula:

n = z^2p(1 − p)w^2

where n is the minimum sample size, w is the estimated error (0.05), p is the estimated HIV prevalence among pregnant women in Sub-Saharan Africa based on systematic reviews and meta-analysis (9%), and z=1.96 by assuming a 95% confidence interval, giving 125 IRIS cases at least needed to answer the study objectives. Considering possible attrition, 5% was added, and the total sample was 133.

Study procedure

Baseline clinical and demographic characteristics were recorded among ART-naïve, HIV-positive pregnant women. The cohort of confirmed HIV-positive pregnant women was initiated on ART as a single population at baseline prior to establishing IRIS incidence. The ART-naïve, HIV-positive pregnant women's active records from the time they were started on ART to week 12 were reviewed and considered in the study, as it is established that the duration of IRIS symptoms is typically two to three months and that they often present within the first four to eight weeks after ART initiation [8]. The ART combination was chosen according to HIV treatment guidelines in pregnancy as guided by the WHO and the clinicians’ case-by-case recommendations.

The clinical teams at study sites prospectively identified IRIS events and collected relevant clinical information that was presented to an endpoint review committee comprising an HIV specialist, a nurse specialist, and a maternal health expert and was meant to ascertain and complement through an independent judgment that, the events were consistent with the AIDS Clinical Trials Group definition criteria for IRIS [9], which includes evidence of ART initiation with resultant increase in CD4 count (≥50 cells/µL or a ≥2-fold rise) and/or virologic suppression (>0.5 log10 decrease in plasma HIV viremia), clinical presentation consistent with an infectious or inflammatory condition, and the absence of an alternative etiology such as the expected course of a previously recognized infection or side effects of medications.

Assessment

General Blood Tests

From the potential female population that would produce IRIS cases for the study after ascertaining HIV-positive status, the HIV test (immunoassay for HIV-1 and HIV-2) was done at the same time as other routine antenatal blood tests (blood group and rhesus factor, full blood count, hepatitis B, rubella, and syphilis) were performed.

History

A complete medical history, including family history, past and current comorbidities, any possible concomitant medicines, and psychosocial history of the current lifestyle, was performed during HIV diagnosis prior to ART initiation.

Gestational Age Estimation

The LMP method was used to assess the estimated age of the pregnancy to ensure it was not above four weeks after conception.

CD4 Cell Counts (HIV Disease Immunology)

This was done on the first visit and then every trimester during pregnancy by obtaining the CD4 absolute count and associated percentages at HIV diagnosis and before ART initiation.

Viral Load Measurement (HIV Disease Virology)

The viral load was assessed at the time of the initial visit, again at the time of starting ART, two weeks after starting ART, and then monthly until complete suppression of the virus was achieved.

Co-infections Assessment

Syphilis serology, sexually transmitted infection screening, tuberculosis chest X-ray, and other comorbidities, including SARS-CoV-2, were assessed based on the WHO guidelines among HIV-infected pregnant women.

Assessment for IRIS Diagnosis

The diagnosis of IRIS was assessed and confirmed in the first 12 weeks after ART initiation. This diagnosis of IRIS was based on the following INSHI criteria: (1) new or worsening infectious or inflammatory symptoms; (2) >1 log decrease in viral load; and (3) the absence of three other explanations (newly acquired infection, predicted course of previously diagnosed infection, and adverse drug effects). Further, experts’ opinions were used to define the IRIS diagnosis based on signs and symptoms to ensure internal validity.

Statistical analysis

A descriptive univariate analysis including WHO-HIV clinical stage, opportunistic infections, maternal age in years, ART combination, maternal body mass index (MBMI), maternal anemia, rhesus factor, parity, and marital status against the cumulative incidence of IRIS and by IRIS type was performed. To calculate the baseline clinical indicators and time to the development of a specific type of IRIS, multivariate Cox regression analysis with IRIS as the outcome was fitted, with censoring effects included prior to 12 weeks after ART initiation using the Survival Package in SPSS [10]. To explore further key relationships between baseline characteristics and IRIS-type diagnosis, a decision tree analysis was performed.

Ethical considerations

Ethical approval was obtained from the University of Nairobi-Kenyatta National Hospital Ethics Committee (ethical approval number: P609/08/2018) before commencing the study. Written informed consent to participate and utilize the active records of eligible ART-naïve, HIV-positive pregnant women at the enrollment stage was obtained. The authority to carry out the research was provided by participating facilities' research management units (Kenyatta National Hospital, reference number: KNH-ERC/RR 305, and Mbagathi Hospital, reference number: MDH/RS/1/VOL 1). The study was conducted in accordance with the principles outlined in the Declaration of Helsinki and local regulatory requirements. Confidentiality and privacy of participants' information were strictly maintained throughout the study.

## Results

A total of 532 HIV-infected, ART-naïve pregnant women were screened. Among them, 143 women diagnosed with IRIS were recruited for the study. After the exclusion of 10 (n=10) participants due to a lack of a definite IRIS diagnosis as well as other critical medical conditions that would compromise the IRIS diagnosis, 133 participants remained. The 133 participants were enrolled at the two sites (91 in Kenyatta National Hospital and 42 in Mbagathi) between June 2019 and May 2020. The majority of women (53%, n=70) were between the ages of 30-39, 51% (n=69), had a normal BMI (18.5-24.9 kg/m^2^), and 80 (60%) were married. Most (50%; n=67) had a parity of two to three. Over 50% (n=73) were at the first stage of HIV infection as determined in WHO guidelines, and 5.3% (n=7) had maternal anemia, with 42% (n=56) presenting with at least a symptom of an opportunistic infection. Tenofovir alafenamide/emtricitabine (TAF/FTC) combination therapy was initiated in 72% (n=96) of the pregnant women treated with ART, while a small number of participants (5.3%; n=11) were put on tenofovir disoproxil fumarate/emtricitabine (TDF/FTC). Of the 133 (25%) participants who experienced IRIS by the 12th week after starting ART, the proportions of IRIS by type were 27.1% (n=36) and 72.9% (n=97) paradoxical and unmasking, respectively (Table 1).

**Table 1:** Baseline characteristics of ART-naïve pregnant women in the first trimester *Abacavir/lamivudine (ABC/3TC) or either tenofovir alafenamide/emtricitabine (TAF/FTC) or tenofovir disoproxil fumarate/emtricitabine (TDF/FTC). Separated/Divorced in the context of this study denotes either not staying together as a married couple or being fully divorced. *** Windowed means the pregnant woman’s husband is dead. ART: antiretroviral therapy; MBMI: maternal body mass index; IRIS: immune reconstitution inflammatory syndrome

Variable		Frequency (%)
WHO-HIV clinical stage	Primary	36 (27.0)
	Stage 1	73 (54.9)
	Stage 2	21 (15.8)
	Stage 3	3 (2.3)
Maternal age (years)	20-29	40 (30.1)
	30-39	70 (52.6)
	40-49	23 (17.3)
ART combination	(ABC/3TC) *	26 (19.5)
	(TAF/FTC)	96 (72.2)
	(TDF/FTC) *	11 (8.3)
MBMI (kg/m^2^)	(<18.5)	21 (15.8)
	(18.5-24.9)	69 (51.9)
	(25 -29.9)	35 (26.3)
	(>30)	8 (6.0)
Hemoglobin level	Abnormal (<11g/dl)	7 (5.3)
	Normal (≥11g/dl)	126 (94.7)
Rhesus factor	Positive	116 (87.2)
	Negative	17 (12.8)
Parity	1	50 (37.6)
	2-3	67 (50.4)
	4-5	14 (10.5)
	>5	2 (1.5)
Marital status	Single	3 (2.25)
	Separated/Divorced**	41 (30.8)
	Married	81 (60.9)
	Windowed***	8 (6.0)
CD4 count (cells/µL)	<500	81 (61.0)
	>500	52 (39.0)
HIV-RNA (log10 copies/mL)	<50	50 (36.0)
	>50	83 (64.0)
IRIS diagnosis	Paradoxical	36 (27.1)
	Unmasking	97 (72.9)

Survival and hazard function at the mean of baseline clinical covariates 

**Figure 1:**
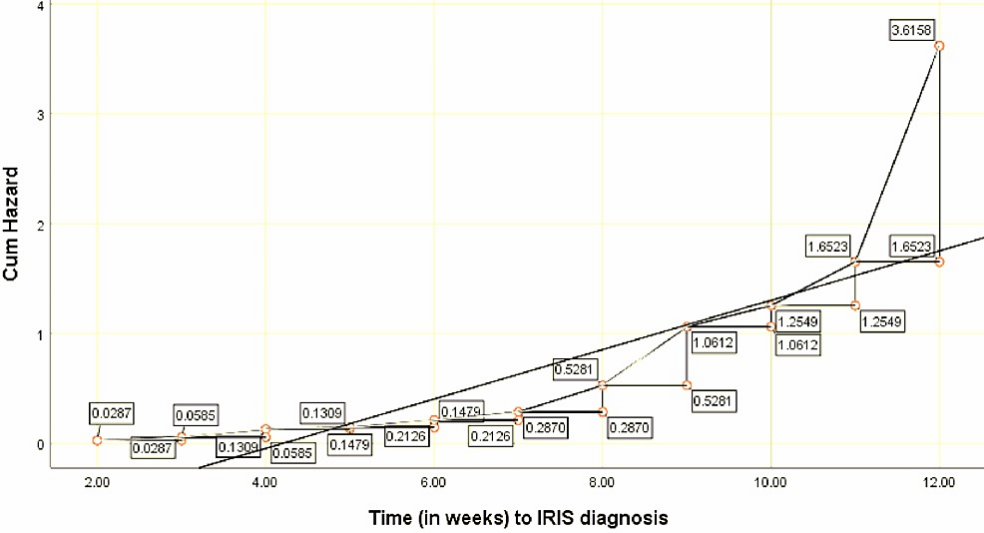
Cumulative hazard for IRIS in general, as shown by hazard functions for the clinical parameters Cum: cumulative; IRIS: immune reconstitution inflammatory syndrome

The total accumulated risk of experiencing IRIS symptoms, as demonstrated by the cumulative hazard function evaluating all the clinical parameters, increased with time, often from the eighth week onwards to the 12th week (Figure 1).

In detail, this shows that, after initiating ART in pregnant women, the rate at which IRIS developed increased gradually with time from week two (hazard ratio (HR)=0.0287) to around week eight; thereafter, drastically towards week 12 (HR=3.6158). This means most IRIS cases, relative to all the clinical parameters, were established after two months of starting ART.

Clinical parameters at the baseline and association with paradoxical or unmasking IRIS

**Figure 2:**
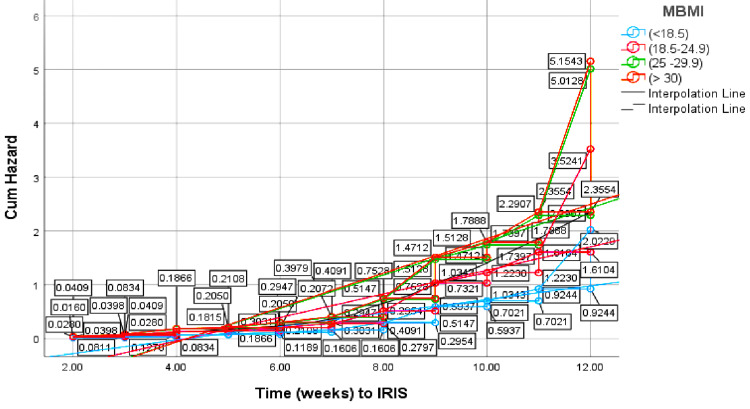
Hazard function for IRIS diagnosis by maternal body mass index (MBMI) Cum: cumulative; IRIS: immune reconstitution inflammatory syndrome

Clinical parameters at baseline following Cox-regression analysis demonstrated that the MBMI of 25-29.9 kg/m^2^ was statistically significant for unmasking IRIS as opposed to paradoxical IRIS, with a positive regression coefficient ((β)=0.907, Wald test (β^) = 6.550, HR=2.478, 95% C.I. 1.237-4.965, P=0.010). Although insignificant, MBMI >30 kg/m^2^ had a seemingly positive regression coefficient towards unmasking IRIS ((β)=0.935, P=0.122). In general, the HR for IRIS increased with an increase in MBMI level, as shown by an increase in the hazard rate (Figure 2).

**Figure 3:**
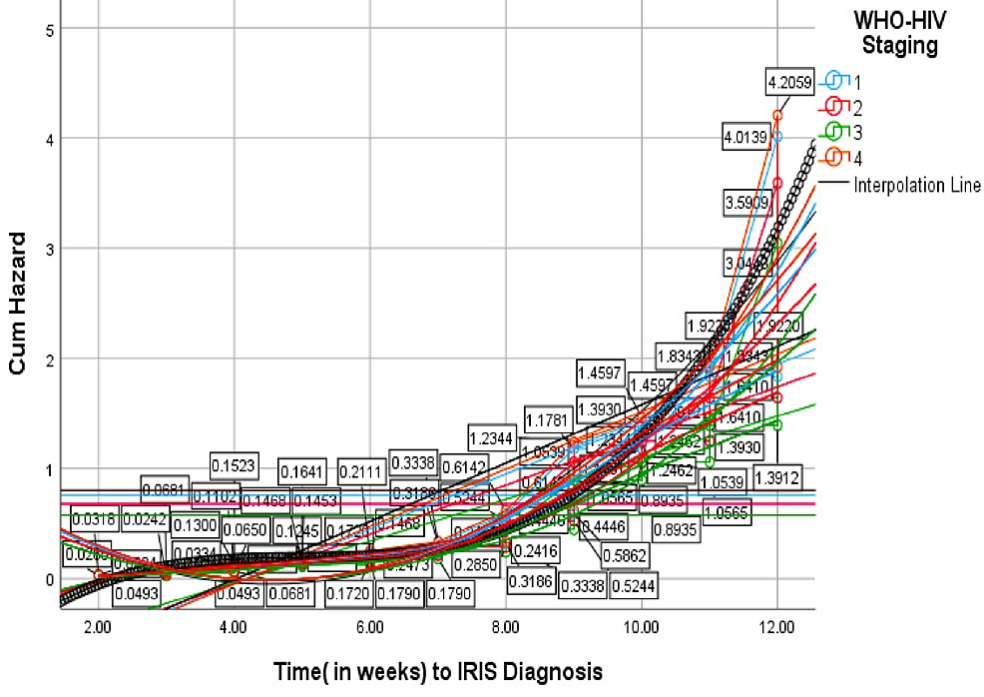
Hazard function for IRIS diagnosis by WHO-HIV clinical staging Cum: cumulative; IRIS: immune reconstitution inflammatory syndrome

The WHO HIV staging (clinical stage 2) and (clinical stage 3) showed negative regression coefficients of (β)=-0.111 and (β)=- 0.276 (P <0.05), respectively, which demonstrates a link towards paradoxical IRIS, although statistically insignificant. The WHO HIV staging (clinical stage 4) was positive with respect to unmasking IRIS, though statistically insignificant ((β)=0.047, HR=1.048, P=0.941). The HR for unmasking IRIS was higher at the primary (stage 1) and clinical stage 4, both with a hazard function of above 4.0 at the end of 12 weeks, as opposed to other stages (Figure 3).

**Figure 4:**
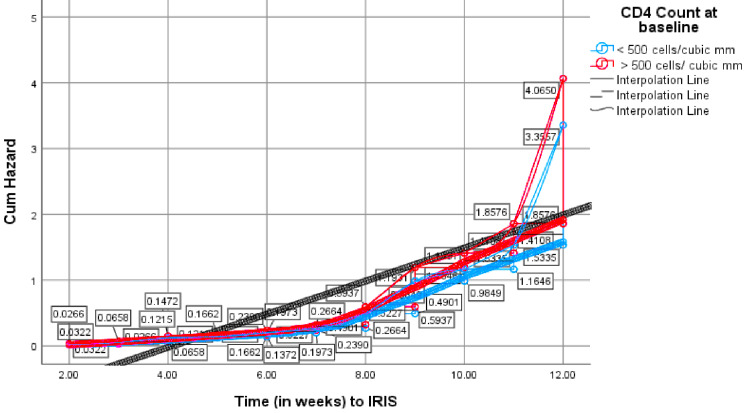
Hazard function for IRIS diagnosis by CD4 count at baseline Cum: cumulative; IRIS: immune reconstitution inflammatory syndrome

**Figure 5:**
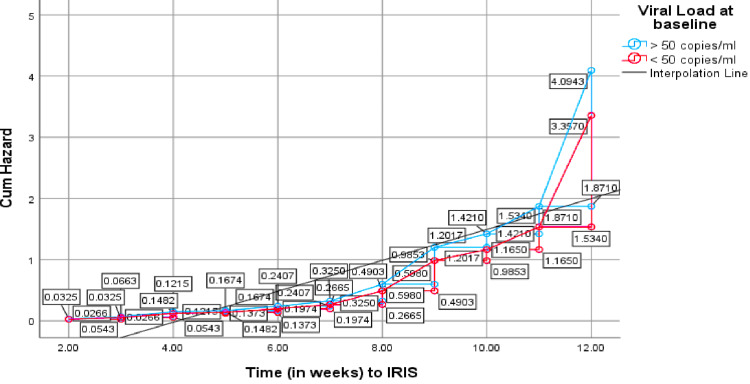
Hazard function for IRIS diagnosis by RNA-viral loads at baseline Cum: cumulative; IRIS: immune reconstitution inflammatory syndrome

A CD4 count of over 500 cells/mm^3 was seemingly associated with an unmasking IRIS diagnosis ((β)=0.192, HR=1.211, P=0.416), while RNA-HIV viral loads above 50 copies/ml were associated with paradoxical IRIS ((β)=- 0.199, HR=0.820, P=0.360). The cumulative hazard for IRIS, in general, was higher in pregnant women with CD4 counts >500 cells/mm^3 and HIV viral loads> 50 copies/mL at baseline, and this risk increased with time towards the 12th week post ART initiation, as shown by higher hazard rates (HR=4.0650 and 4.0943), respectively (Figures 4-5).

All the above specific clinical parameters are presented herein, demonstrating that MBMI of 25-29.9 kg/m^2^, WHO-HIV clinical stage 4, CD4 count of over 500 cells/mm^3, and a parity above five were linked with the diagnosis of unmasking IRIS, while WHO-HIV clinical stage 2, stage 3, and RNA-HIV viral loads above 50 copies/ml were linked with paradoxical IRIS. This result suggests different clinical parameters among ART-naïve, HIV-infected women predict a specific type of IRIS, which could be due to the immunopathology of HIV infection coupled with pregnancy pro-inflammatory responses in the first trimester. Precisely, advanced HIV infection, above the minimum level of CD4 count in cells/mm^3, and increasing MBMI predict a higher proportion of IRIS, as 73% was the unmasking type (Table 2).

**Table 2:** Cox-model regression analysis for prognostic clinical parameters (variables) on paradoxical or unmasking IRIS outcome at baseline. A positive coefficient (β) favors unmasking IRIS, while a negative one favors paradoxical IRIS. The Wald test (also called the Wald chi-squared test) is a way to find out if explanatory variables in a model are significant. Exp (β) is the predicted change in odds for a unit increase in the predictor.  Standard error (SE) is a statistical term that measures the accuracy with which a sample distribution represents a population by using the standard deviation. MBMI: maternal body mass index; B: unstandardized beta; df: degrees of freedom; Sig.: significance; Exp(B): exponential value of B; IRIS: immune reconstitution inflammatory syndrome

Variables in the equation							
Variable	B	SE	Wald	df	Sig.	Exp(B)	95.0% CI for Exp(B)
							Lower-Upper
MBMI (< 18.5)			7.031	3	.071		
MBMI (18.5-24.9)	.555	.329	2.843	1	.092	1.742	.914-3.321
MBMI (25 -29.9)	.907	.355	6.550	1	.010	2.478	1.237-4.965
MBMI (> 30)	.935	.605	2.394	1	.122	2.548	.779 -8.333
Rhesus factor	-.332	.405	.673	1	.412	.718	.325-1.586
Parity 1			5.981	3	.113		
Parity 2-3	-.542	.260	4.350	1	.037	.582	.349-.968
Parity 3-4	-.163	.340	.229	1	.632	.850	.437-1.654
Parity >5	.743	.773	.925	1	.336	2.103	.462-9.570
WHO-HIV Staging: Stage 1			.700	3	.873		
WHO-HIV Staging: Clinical 2	-.111	.267	.173	1	.677	.895	.530-1.511
WHO-HIV Staging: Clinical 3	-.276	.345	.644	1	.422	.758	.386-1.490
WHO-HIV Staging: Clinical 4	.047	.632	.005	1	.941	1.048	.304-3.614
CD4 count	.192	.236	.663	1	.416	1.211	.763-1.922
Viral load	-.199	.217	.838	1	.360	.820	.536-1.254

Decision tree analysis on prediction of IRIS incidence 

**Figure 6:**
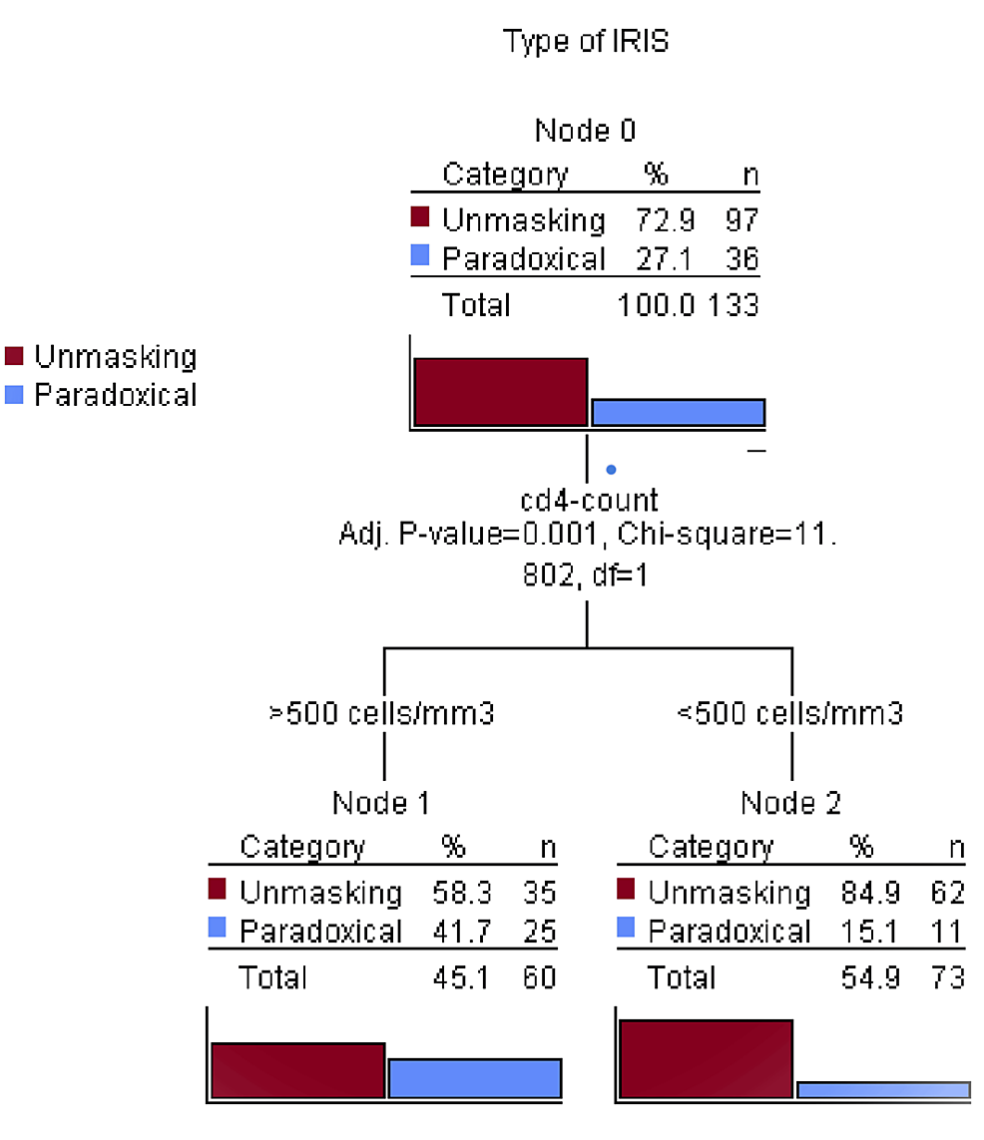
Decision tree analysis showing the proportions of unmasking paradoxical IRIS by baseline CD4 counts CD4 is in cell/mm^3; Adj. stands for adjusted; "Node" means an outcome on a tree diagram; IRIS: immune reconstitution inflammatory syndrome

To assess the predictions of IRIS type by the 12th week post ART initiation for exploratory and confirmatory classification, decision tree analysis was performed by including all the clinical parameters and demographic characteristics of the participants, including location, education, occupation, religion, marital status, income source, baseline CD4 count and HIV-RNA viral loads, MBMI, co-infections, and maternal age. Using the chi-squared automatic interaction detection (CHAID) method, baseline CD4 count was the best predictor of IRIS type. On this, 85% (P=0.001) of the ART-naïve pregnant women with <500 cells/mm^3 were diagnosed with unmasking IRIS, compared to 15.1% (n=11) of those diagnosed with paradoxical IRIS. Similarly, 58% (n=35) of women with >500 cells/mm^3 at baseline were diagnosed with unmasking IRIS compared to those diagnosed with paradoxical IRIS (42%, n=25). This shows that an unmasking IRIS diagnosis was predicted by baseline CD4 count levels more than paradoxical IRIS (Figure 6). 

**Figure 7:**
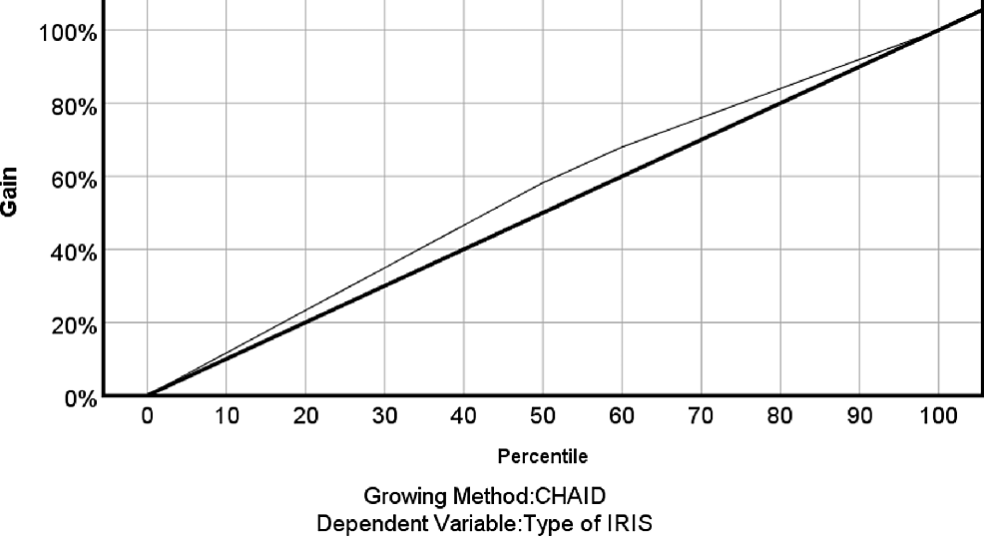
The gain chart curve on the prediction model of IRIS Chi-squared automatic interaction detection (CHAID) is a statistical technique mainly used to understand the characteristics that are most associated with a given outcome or group membership. IRIS: immune reconstitution inflammatory syndrome

The gain chart shown in Figure 7 demonstrates a gain greater than one, indicating that the results from the predictive model (the curved line) are better than those from the random model (the straight line).

**Figure 8:**
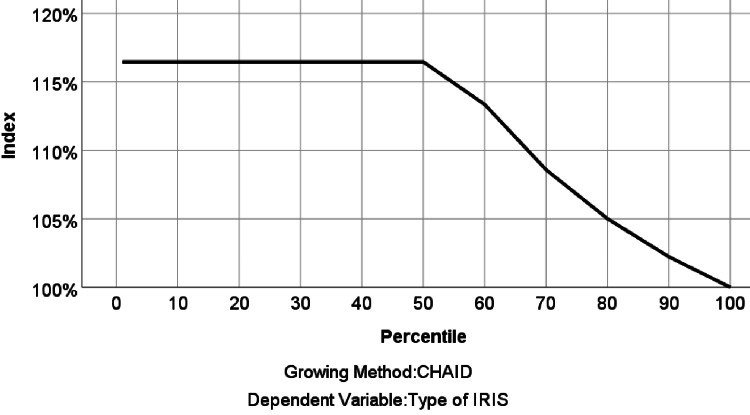
The index chart shows the ratio of the node response percentage. Chi-square automatic interaction detector (CHAID) analysis builds a predictive model, or tree, to help determine how variables best merge to explain the outcome in the given dependent variable. IRIS: immune reconstitution inflammatory syndrome

This indicated a plausible prediction, with approximately 60% of the data accounting for approximately 70% of the true unmasking IRIS cases. The index (lift) chart measures how much better can be expected with the predictive model compared to the random model. The index value starts well above 100% (at about 117%), showing an increase above the reference line (random model line) that gradually drops off, complementing the gain chart as shown in Figure 8.

## Discussion

In this prospective, observational, active records-based study, we evaluated IRIS incidence by type and predictors, including survival and hazard risk analysis using the Cox model.

Unmasking IRIS was more common in this population of ART-naïve pregnant women as compared to paradoxical IRIS, similar to a study that reported 69.4% unmasking and 27.8% paradoxical IRIS [11]. This was further within the percentage range of 59% to 76% obtained from other studies [12]. Another study reports a 22% paradoxical IRIS [13], which is close to the current proportion obtained from this study.

An MBMI of 25-29.9 kg/m^2^ clearly predicted unmasking IRIS, as it has also been depicted in a study that established a BMI ≥20 kg/m^2^ as a probable predictor of IRIS [14]. By contrast, another study established a BMI <18.5 kg/m^2^ (HR=2.15, 95% CI 1.07-4.3, p=0.03) as an IRIS diagnosis predictor. A parity above five clearly predicted unmasking IRIS, and although there are no clear studies elucidating this fact, one study concluded that gestational age and a parity above four influenced T lymphocyte subset levels [15], a key phenomenon in IRIS development. The WHO-HIV staging (clinical stage 4) was positive for unmasking IRIS, and as it was the common form of IRIS, these findings are elucidated in studies that report on HIV late presenters [16]. Again, another study shows that risk factors for IRIS include an advanced state of immunosuppression and disseminated opportunistic infections at ART initiation [17], factors clearly associated with advanced HIV infection. The hazard risk for unmasking IRIS was higher at primary and clinical stage 4. This is matched in a study that described five patients with advanced HIV-1 infection in whom initiation of highly active antiretroviral therapy (HAART) resulted in the unmasking of an underlying occult opportunistic infection [18]. An unmasking IRIS diagnosis can be attributed to the fact that people with acute HIV infection may experience fever, lymphadenopathy, pharyngitis, skin rash, myalgia, arthralgia, and other symptoms [19].

In contrast to other studies that showed a higher risk of IRIS among people starting treatment with a very low CD4 cell count (usually below 100 cells/mm^3) [20], the current study established that a CD4 count of over 500 cells/mm^3 is associated with an unmasking IRIS diagnosis in this ART-naïve cohort of pregnant women. This may be due to pregnancy-related proinflammatory factors. Again, the already-high CD4 count before ART initiation may imitate the known increase of the same that is associated with developing IRIS [21]. Moreover, IRIS can occur at any CD4 count [22].

On the other hand, RNA-HIV viral loads >50 copies/mL showed a positive direction with paradoxical IRIS, similar to existing research [23]. However, on the contrary, some reports have shown that it is the dramatic reduction of RNA-HIV viral load levels after starting ART that significantly demonstrates IRIS development [24]. This concept may be justified by the evidence that a pre-existing latent opportunistic infection with a high antigenic burden at the time of starting ART increases the risk and severity of IRIS [13]. In a real sense, paradoxical IRIS is linked with the most opportunistic infection as compared to unmasking IRIS [25]. The cumulative hazard risk for IRIS was higher in pregnant women with both a higher baseline CD4 count and viral loads that increased towards the 12th week post ART initiation. This concurs with findings that showed an increased baseline CD4+ T cell count among women of reproductive age on ART [26], while other studies found higher viral loads [27]. The increased risk for IRIS with time as per this study is also reflected in previous studies [20].

Decision tree analysis demonstrated baseline CD4 count levels as the best predictor of IRIS. Notably, ART-naïve pregnant women with a CD4 count below 500 cells/mm^3 as opposed to those with a CD4 count above 500 cells/mm^3 were diagnosed with unmasking IRIS compared to those diagnosed with paradoxical IRIS. This finding is supported by a study conducted in an East African context that found patients with a lower pre-ART CD4 count had a higher chance of developing IRIS in general [28], similar to another study that showed patients who developed IRIS had lower baseline CD4 counts [29]. This is by noting that unmasking IRIS was the most common, compared with paradoxical IRIS, at a proportion of over two-thirds in the current findings.

This study had several limitations. First, the identification of IRIS by type, either paradoxical or unmasking, may have been compromised due to the thin gap between the two; however, the clear approach was based on and guided by specific biomarkers such as CD4 count and RNA-viral loads at baseline. Again, the duration of time a woman stayed after contracting HIV may not have been known before conceiving or after. However, based on the clinical parameters guiding HIV infection staging by the WHO, it was possible to estimate the same. Last, due to the slow development of disease after being infected with HIV, some pregnant women may have used medications without clinical directives, thereby compromising the process of IRIS identification. This issue, however, was tackled by using a combined guideline to diagnose IRIS, and, as such, this limitation was somewhat mitigated. Despite these limitations, we believe the study results provide valuable information about the incidence of IRIS by type, whether unmasking or paradoxical. These results estimate the survival time for the event of either IRIS in general or IRIS by type while accounting for key baseline clinical parameters predicting IRIS in ART-naïve pregnant women.

## Conclusions

Unmasking was found to be the most common type of IRIS among the population of ART-naïve pregnant women predicted independently, primarily by a baseline CD4 count of below 500 cells/mm^3 and HIV clinical infection stages 1 and 4. An MBMI of 25-29 kg/m^2^ and baseline viral loads above 50 copies/mL predicted paradoxical IRIS. These findings suggest a CD4 count below 500 cells/mm^3, early and later stages of HIV infection may work together to aid highly in the development of IRIS, especially the unmasking type. Further research should validate the potential effect of MBMI, maternal age, HIV baseline biomarkers, and HIV infection clinical stage in predicting unmasking or paradoxical IRIS and re-affirm the evidence regarding survival time to IRIS diagnosis among ART-naïve pregnant women by ART combination.
